# Co-regulation of the Notch and Wnt signaling pathways promotes supporting cell proliferation and hair cell regeneration in mouse utricles

**DOI:** 10.1038/srep29418

**Published:** 2016-07-20

**Authors:** Jingfang Wu, Wenyan Li, Chen Lin, Yan Chen, Cheng Cheng, Shan Sun, Mingliang Tang, Renjie Chai, Huawei Li

**Affiliations:** 1 Otorhinolaryngology Department of Affiliated Eye and ENT Hospital, State Key Laboratory of Medical Neurobiology, Fudan University, Shanghai, 200031, PR China; 2 Institutes of Biomedical Sciences, Fudan University, Shanghai, 200032, PR China; 3 Central laboratory, Affiliated Eye and ENT Hospital of Fudan University, Shanghai, 200031, PR China; 4 Key Laboratory of Hearing Medicine of National Health and Family Planning Commission, Shanghai, 200031, PR China; 5 MOE Key Laboratory of Developmental Genes and Human Disease, State Key Laboratory of Bioelectronics, Institute of Life Sciences, Southeast University, Nanjing 210096, PR China.; 6 Co-innovation Center of Neuroregeneration, Nantong University, Nantong, 226001, PR China

## Abstract

This work sought to determine the crosstalk between the Notch and Wnt signaling pathways in regulating supporting cell (SC) proliferation and hair cell (HC) regeneration in mouse utricles. We cultured postnatal day (P)3 and P60 mouse utricles, damaged the HCs with gentamicin, and treated the utricles with the γ-secretase inhibitor DAPT to inhibit the Notch pathway and with the Wnt agonist QS11 to active the Wnt pathway. We also used Sox2-CreER, Notch1-flox (exon 1), and Catnb-flox (exon 3) transgenic mice to knock out the Notch pathway and activate the Wnt pathway in Sox2+ SCs. Notch inhibition alone increased SC proliferation and HC number in both undamaged and damaged utricles. Wnt activation alone promoted SC proliferation, but the HC number was not significantly increased. Here we demonstrated the cumulative effects of Notch inhibition and Wnt activation in regulating SC proliferation and HC regeneration. Simultaneously inhibiting Notch and overexpressing Wnt led to significantly greater SC proliferation and greater numbers of HCs than manipulating either pathway alone. Similar results were observed in the transgenic mice. This study suggests that the combination of Notch inhibition and Wnt activation can significantly promote SC proliferation and increase the number of regenerated HCs in mouse utricle.

Hair cells (HCs) of the inner ear sensory epithelium are highly differentiated. The mammalian utricle, which is a vestibular organ that requires HCs to detect linear acceleration, has limited capacity for spontaneous HC regeneration. However, when the inner ear sensory HCs have been damaged by ototoxic drugs, acoustic trauma, or genetic defects, the quantity and quality of the spontaneously regenerated HCs is not sufficient to recover vestibular function^1^. Inducing the sensory precursor cells to divide and trans-differentiate into new HCs might be an ideal way to rescue balance dysfunction after damage to the inner ear.

In birds, the utricle has the capacity to fully regenerate lost HCs after damage to the sensory epithelium^2^,^3^. This HC regeneration consists mainly of mitotic regeneration – in which supporting cells (SCs) proliferate first and then differentiate into HCs – and direct trans-differentiation – in which SCs directly differentiate into HCs without first entering the cell cycle^4^,^5^. In contrast to the robust HC regeneration capacity of birds, the mammalian utricle exhibits very little proliferation after HC damage in adults^6^,^7^,^8^,^9^,^10^,^11^,^12^,^13^,^14^. Multiple studies have reported that SCs can serve as a reliable source to regenerate HCs, and during HC regeneration the HC number increases while the SC number decreases^6^,^8^,^11^,^12^,^13^. However, in the utricle sensory epithelium, HCs are interdigitated by SCs, and the loss of SCs will also lead to the death of the newly differentiated HCs^15^. Thus, exhausting the supply of SCs to regenerate HCs is not an effective strategy for long-term HC regeneration. Previous studies showed that after HC damage SCs can reenter the cell cycle and can be labeled with mitotic tracers^6^,^9^,^10^,^16^; thus it is possible to promote the proliferation of SCs first and then let the proliferated SCs differentiate into HCs, which could preserve the SC number while giving rise to new HCs. However, in previous studies the majority of regenerated HCs come from direct trans-differentiation, and mitotic HC regeneration only provides a small portion of the newly regenerated HCs.

Cells respond to various intracellular and extracellular signals, and cell signal transduction is a fundamental biological activity. Many signaling pathways are involved in inner ear development and sensory HC regeneration, and there is crosstalk between different signaling pathways^17^,^18^,^19^,^20^,^21^. These pathways interact with each other and collaboratively control sensory progenitor cell division and differentiation in the inner ear. Therefore, the ability to manipulate the crosstalk between signaling pathways will be required for inducing sensory progenitor cells to proliferate and differentiate so as to mitotically regenerate HCs in the mammalian inner ear.

The Notch and Wnt signaling pathways are highly conserved signaling pathways found in various organs, including the inner ear sensory epithelium. During the development of the inner ear, Notch signaling induces HC formation through lateral inhibition and results in the delicate mosaic pattern seen in the auditory sensory epithelium^22^,^23^. The inhibition of Notch signaling leads to the generation of numerous ectopic HCs at the expense of SCs^8^,^24^,^25^,^26^,^27^. Wnt signaling plays a dual role in regulating the inner ear stem/progenitor cells by stimulating both the expansion and differentiation of HC progenitors during inner ear development^28^,^29^,^30^,^31^,^32^. Previous studies reported that inhibiting Notch signaling could up-regulate Wnt signaling, while inhibiting Wnt signaling could down-regulate Notch signaling^19^,^20^,^33^, and this suggested that the Notch and Wnt signaling pathways interact with each other. However, the crosstalk between Notch and Wnt signaling in regulating SC proliferation and HC regeneration remains unknown.

In this study, we took advantage of a pharmaceutical agonist and antagonist as well as transgenic mice to investigate the effects of simultaneous Notch inhibition and Wnt activation in controlling the proliferation of SCs and regeneration of HCs with or without HC damage. We found that Notch inhibition induced SC proliferation and mitotic HC regeneration, while Wnt activation significantly enhanced the SC proliferation and mitotic HC regeneration induced by Notch inhibition, thus Notch inhibition and Wnt activation have cumulative effects in regulating SC proliferation and HC regeneration. These findings have improved our understanding of the crosstalk between the Notch and Wnt pathways and might provide a new method for promoting mammalian HC regeneration after injury to the sensory epithelium.

## Results

### Simultaneous Notch inhibition and Wnt activation induces SC proliferation in neonatal mouse utricles *in vitro*

**Figure 1:**
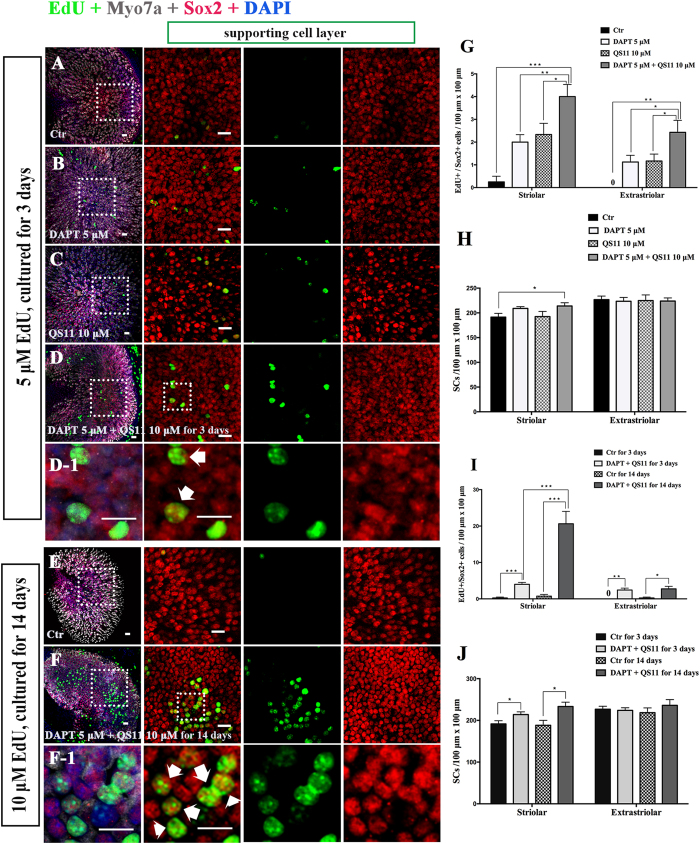
The P3 mouse utricles were cultured with DAPT, QS11, or a combination of DAPT and QS11 for 3 days or 14 days. (Scale bar = 10 μm) **(A)** The untreated P3 mouse utricles cultured for 3 days showed very few EdU+/Sox2+ cells in the sensory epithelium. **(B)** When utricles were treated with DAPT for 3 days, there were some EdU+/Sox2+ cells in the sensory epithelium. **(C)** When utricles were treated with QS11 for 3 days, there were few EdU+/Sox2+ cells in the sensory epithelium and the number of SCs did not change significantly (*p* > 0.05). **(D)** In the utricles treated with a combination of DAPT and QS11 for 3 days, there were more EdU+/Sox2+ cells compared to the DAPT-only and the QS11-only group (*p* < 0.05). The number of SCs in the striolar region was greater than the control group (*p* < 0.05). **(D-1)** The high magnifications of image D show two of EdU+/Sox2+ cells in the utricles. **(G)** The histograms show differences in the number of EdU+/Sox2+ cells between the groups cultured for 3 days. **(H)** The histograms show differences in the number of SCs between the groups cultured for 3 days. **(E)** The untreated P3 mouse utricles cultured for 14 days showed very few EdU+/Sox2+ cells in the sensory epithelium. **(F)** In the cultured utricles co-treated with DAPT and QS11 and supplied with 10 μM EdU, there were many EdU+/Sox2+ cells in the utricles and a large number of Sox2+ SCs. **(F-1)** The EdU+/Sox2+ cells in the utricles are shown at high magnification. **(I)** The number of EdU+/Sox2+ SCs in the 14-day culture was significantly greater than in the 3-day cultured. **(J)** The histograms show differences in the number of SCs between the utricles co-treated with DAPT+QS11 for 3 days and 14 days. The cells were counted per 100 μm × 100 μm in the striolar or extrastriolar region of the utricles. (*p < 0.05, **p < 0.01, ***p < 0.001).

Previous studies revealed that membrane-bound Notch physically associates with unphosphorylated active β-catenin in stem cells and cancer cells and that Notch signaling negatively regulates the post-translational accumulation of active β-catenin protein^34^,^35^. Our own reports also demonstrated that Notch inhibition can induce SC proliferation and mitotic HC regeneration via activation of Wnt signaling. However, the role of interaction between Notch and Wnt signaling in regulating SC proliferation and HC regeneration remains unknown in the mouse utricle. To investigate the effect of co-regulation of Notch and Wnt signaling on the cultured vestibular sensory epithelium, postnatal day (P)3 mouse utricles were dissected out and treated with 5 μM of the γ-secretase inhibitor DAPT and/or 10 μM of the β-catenin nuclear translocation agonist QS11 for 72 hours. The cell proliferation maker EdU (5 μM) was administered together with the DAPT and QS11 for 24 hours. After three days, the cultured utricles were stained with antibodies against Myo7a and Sox2. In the utricles treated with 5 μM DAPT, there were some EdU+/Sox2+ cells, especially in the striolar region (Fig. 1B,E, Supplemental Table 2). In the QS11-treated utricles, there were also some EdU+/Sox2+ cells (Fig. 1C,E, Supplemental Table 2). In the DAPT and QS11 co-treated utricles, there were significantly more EdU+/Sox2+ cells (Fig. 1D,G, Supplemental Table 2) compared with the utricles treated with DAPT or QS11 alone (Fig. 1G, Supplemental Table 2, *p* < 0.05), and the SC number in the striolar region was also slightly increased compared to with control (Fig. 1H, Supplemental Table 2, *p* < 0.05).

In order to label as many as the proliferated cells as possible, in a separate experiment we cultured the utricle for a longer period with more frequent and concentrated EdU exposure. We treated the cultured utricles with 5 μM DAPT and 10 μM QS11 for 14 days and provided 10 μM EdU every day. Compared with the control group, we found significantly more EdU+/Sox2+ cells in co-treated group, and most of them were located in the striolar region (Fig. 1F,I, Supplemental Table 2, *p* < 0.05). Furthermore, compared with the 3-day treatment with DAPT and QS11, we found that significantly more EdU+/Sox2+ cells were identified in both the striolar and extrastriolar regions in 14-day treatment group (Fig. 1I, Supplemental Table 2, *p* < 0.05). These results show that simultaneous Notch inhibition and Wnt activation can significantly induce SC proliferation in neonatal mouse utricles, and this demonstrates the cumulative effects of Notch inhibition and Wnt activation in promoting the proliferation of SCs in the mouse utricle.

### Simultaneous Notch inhibition and Wnt activation induces mitotic regeneration of HCs and increases the HC number in neonatal mouse utricles *in vitro*

Similar to the above experiments, we cultured the utricles from P3 mice and treated them with 5 μM DAPT and/or 10 μM QS11 for 72 hours. We treated all cultures with EdU (5 μM) was for the first 24 hours and then stained them with antibodies against Myo7a and Sox2.

**Figure 2:**
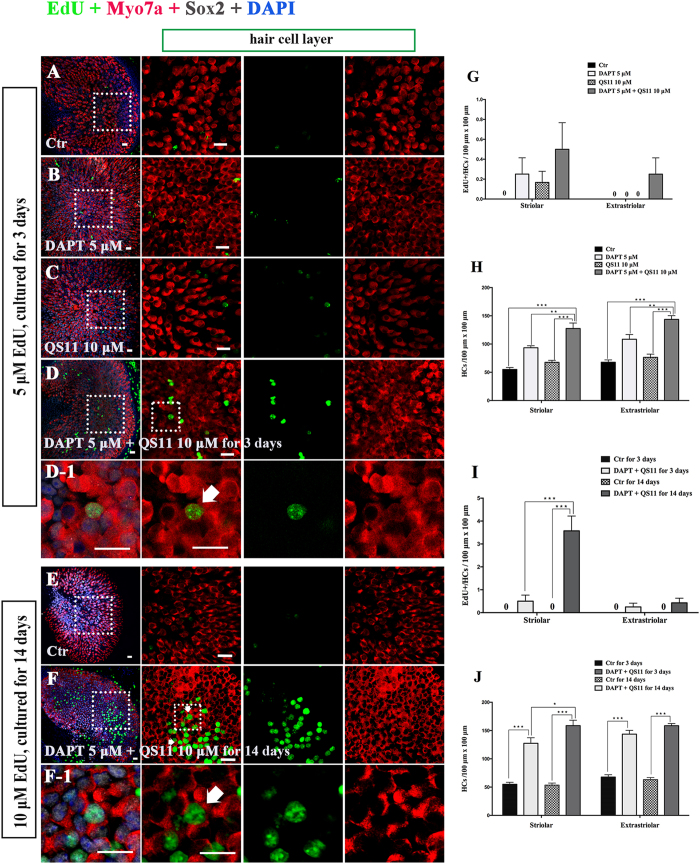
The P3 mouse utricles were cultured with DAPT, QS11, or a combination of DAPT and QS11 for 3 days or for 14 days. (Scale bar = 10 μm) **(A)** The untreated P3 mouse utricles cultured for 3 days. **(B)** When utricles were treated with DAPT for 3 days, there were few EdU+/Myo7a+ cells in the sensory epithelium and the number of HCs increased (*p* < 0.05 vs. the control). **(C)** When the utricles were treated with QS11 for 3 days, there were few EdU+/Myo7a+ cells in the sensory epithelium and the number of HCs did not change significantly (*p* > 0.05). **(D)** In the DAPT+QS11 combination-treated utricles, there were some EdU+/Myo7a+ cells in the utricles, which were mostly in the striolar region. There were more HCs than the other groups (*p* < 0.05). **(D-1)** The high magnifications of the image in (**D**) show the EdU+/Myo7a+ cell in the utricle. **(G)** The histograms show differences in the number of EdU+ HCs between these groups cultured for 3 days. **(H)** The histograms show the differences in the number of HCs between these groups cultured for 3 days. **(E)** The untreated P3 mouse utricles cultured for 14 days. **(F)** In the utricles treated with 10 μM EdU and a combination of DAPT and QS11 for 14 days, there were some EdU+/Myo7a+ cells, which were found mostly in the striolar region. The number of HCs in the utricles had clearly increased. **(F-1)** The high magnifications of image F show EdU+/Myo7a+ cells in the utricles. **(I)** In the cultured utricles co-treated with DAPT and QS11 and supplied with 10 μM EdU for 14 days, the number of EdU+/Myo7a+ cells was significantly increased compared with the utricles co-treated for 3 days. **(J)** The histograms show the differences in the number of HCs between the utricles co-treated with DAPT + QS11 for 3 days or 14 days. The cells were counted per 100 μm × 100 μm in the striolar or extrastriolar region of the utricles (*p < 0.05, **p < 0.01, ***p < 0.001).

In the utricles treated with 5 μM DAPT, we found that the number of HCs was increased compared to controls (Fig. 2B,H, Supplemental Table 2, *p* < 0.05), but few EdU+/Myo7a+ cell were identified.

In QS11-treated utricles, we found no significant change in the number of HCs compared with controls (Fig. 2C,H, Supplemental Table 2, *p* > 0.05), and we also observed few EdU+/Myo7a+ cells. In the DAPT and QS11 co-treated utricles, we found that the number of HCs was significantly increased compared with the control group as well as the DAPT/QS11-only groups (Fig. 2D,H, Supplemental Table 2, *p* < 0.05), and the co-treated group also contained some EdU+/Myo7a+ cells, especially in the striolar region (Fig. 2D,G, Supplemental Table 2).

In order to trace the proliferated supporting cells, we cultured the utricles with 5 μM DAPT and 10 μM QS11 for 14 days and supplied them with 10 μM EdU every day. We observed significantly more EdU+/Myo7a+ cells in the co-treated group compared with the control group, and most of the EdU+/Myo7a+ cells were located in the striolar region (Fig. 2F,I, Supplemental Table 2, *p* < 0.05). Furthermore, compared with the results from the 3-day co-treatment, the number of EdU+/Myo7a+ HCs as well as the number of Myo7a+ HCs was significantly increased after 14 days of co-treatment (Fig. 2F,I,J, Supplemental Table 2, *p* < 0.05). These results demonstrated the cumulative effects of Notch inhibition and Wnt activation in regulating mitotic HC generation.

### After HC ablation, simultaneous Notch inhibition and Wnt activation promotes SC proliferation in neonatal mouse utricles *in vitro*

The utricles were dissected out from P3 wild-type mice and cultured with 2 mM gentamicin for 48 hours to damage the HCs. We then treated the utricles with 5 μM DAPT and/or 10 μM QS11 for another 7 days.

**Figure 3:**
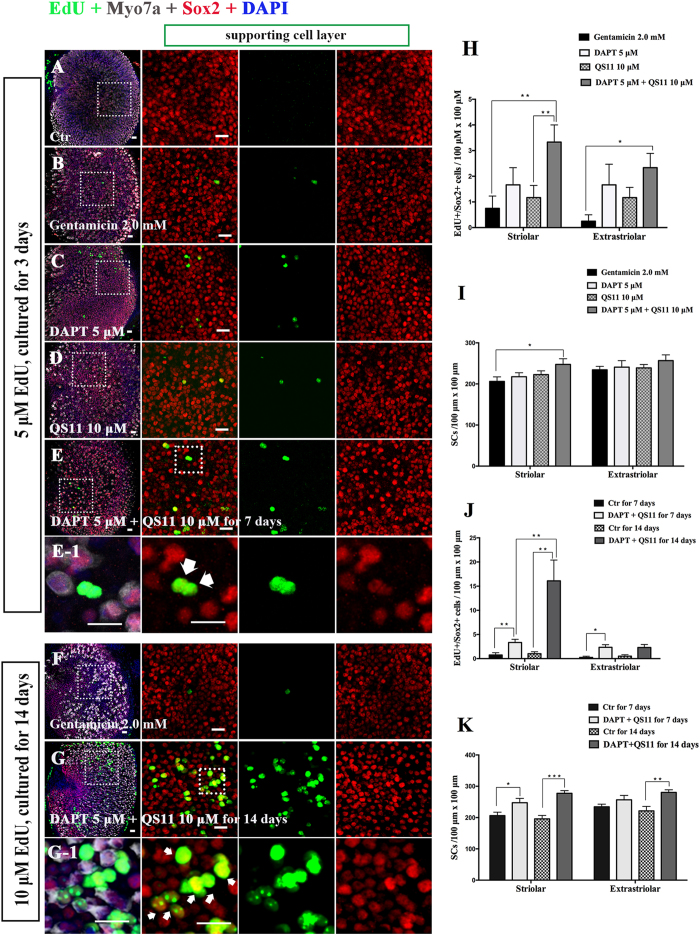
The P3 mouse utricles were treated with gentamicin for 48 hours then treated with DAPT and QS11 separately or in combination for 7 days or for 14 days. (Scale bar = 10 μm) (**A**) The untreated P3 mouse utricles cultured for 7 days. (**B**) The P3 mouse utricles were treated with gentamicin for 48 hours and then cultured for another 7 days. (**C**) When utricles were treated with DAPT for 7 days after being treated with gentamicin, there were some EdU+/Sox2+ cells in the sensory epithelium. (**D**) When utricles were treated with QS11 for 7 days after being treated with gentamicin, there were few EdU+/Sox2+ cells in the sensory epithelium. (**E**) When utricles were treated with a combination of DAPT+QS11 for 7 days after being treated with gentamicin, there were more EdU+/Sox2+ cells in the sensory epithelium. (**E-1**) The high magnifications of picture E show EdU+/Sox2+ cells in the utricles. (**H**) The histograms show differences in the number of EdU+/Sox2+ cells between these groups cultured for 7 days. (**I**) The histograms show differences in the number of SCs between these groups cultured for 7 days. **(F)** The P3 mouse utricles were treated with gentamicin for 48 hours and cultured for 14 days. The HCs were clearly damaged. **(G)** In the cultured utricles co-treated with DAPT and QS11 and supplied with 10 μM EdU, there were many more EdU+/Sox2+ cells in the utricles compared with the utricles co-treated for 7 days. **(G-1)** The high magnifications of picture G show EdU+/Sox2+ cells in the utricles. **(J)** The histograms show that the number of EdU+/SCs in the 14-day culture group was significantly greater than in the 7-day culture group. **(K)** The histograms show the differences in the number of SCs between the utricles co-treated with DAPT+QS11 for 7 days and 14 days. The cells were counted per 100 μm × 100 μm in the striolar or extrastriolar region of the utricles. (*p < 0.05, **p < 0.01, ***p < 0.001)

In the gentamicin-treated group, there were few EdU+/Sox2+ SCs among the sensory epithelium of the utricles. In the 5 μM DAPT-treated groups, there were some EdU+/Sox2+ SCs in the sensory epithelium (Fig. 3C,H, Supplemental Table 3). In the QS11-treated groups, we also found some EdU+/Sox2+ SCs in the sensory epithelium (Fig. 3D,H, Supplemental Table 3). In the DAPT and QS11 co-treated group, we found significantly more EdU+/Sox2+ cells compared with than the control and QS11-only treated groups (*p* < 0.05) and slightly more EdU+/Sox2+ cells compared with the DAPT-treated group (Fig. 3E,H, Supplemental Table 3). The overall number of Sox2+ SCs in the striolar region was also increased compared with controls (Fig. 3I, Supplemental Table 3, *p* < 0.05).

In order to label as many proliferated SCs as possible, we cultured the utricles with 5 μM DAPT and 10 μM QS11 for 14 days after HC loss induced by gentamicin and supplied them with 10 μM EdU every day. We identified many more EdU+/Sox2+ cells in the striolar region of the cultured utricles (Fig. 3G,J, Supplemental Table 3). Compared with the results from the 7-day co-treatment, the numbers of EdU+/Sox2+ SCs and Sox2+ SCs were significantly increased after 14 days of co-treatment with DAPT and QS11 (Fig. 3G,J,K, Supplemental Table 3, *p* < 0.05).

In order to further study the effects of simultaneous Notch inhibition and Wnt activation, we took advantage of transgenic mice and mated the Sox2-CreER mice with Notch1-flox (exon1) and Catnb-flox (exon3) mice. The P3 pups were genotyped before they were sacrificed, and the utricles were then dissected out and cultured with 2 mM gentamicin for 48 h. After this culture period, 4OH-tamoxifen was added to the culture medium to activate the Cre recombinase and the utricles were cultured for another 7 days.

**Figure 4:**
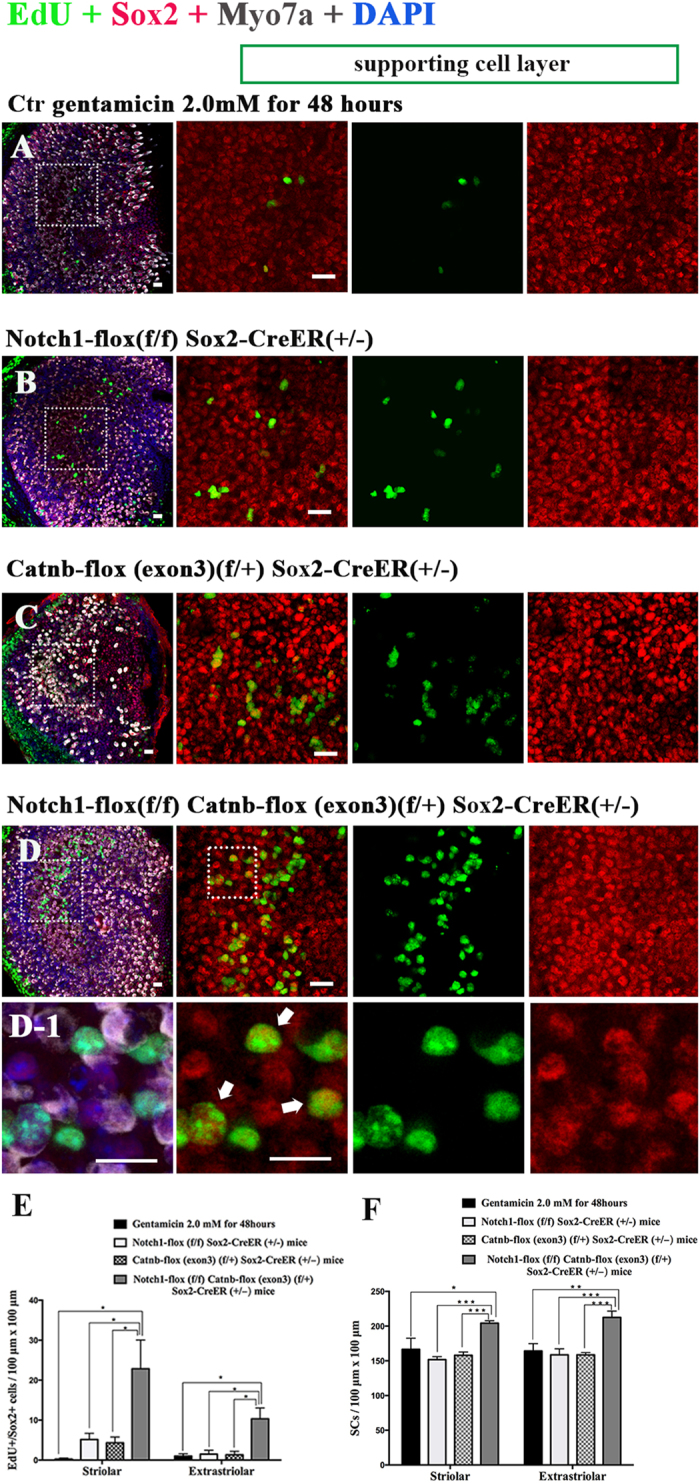
Sox2-CreER and Notch1-flox (exon 1) mice were mated with Catnb-flox (exon 3) mice to generate pups. The P3 mouse utricles were dissected and cultured with 2 mM gentamicin for 48 hours to damage the HCs of the utricles. The cultured utricles were treated with tamoxifen and cultured for an additional 7 days. (Scale bar = 10 μm) (**A**) In the control group, there were no obvious EdU+/Sox2+ cells in the sensory epithelium of the utricles. (**B**) In the Notch1-flox (f/f) Sox2-CreER (+/−) mouse utricles, there were some EdU+/ Sox2+ cells in the sensory epithelium. (**C**) In the Catnb-flox (exon3) (f/+) Sox2-CreER (+/−) mouse utricles, there were few EdU+/Sox2+ cells in the sensory epithelium of the utricles. (**D**) In the Notch1-flox (f/f) Catnb-flox (exon3) (f/+) Sox2-CreER (+/−) group, there were significantly more EdU+/Sox2+ cells in the sensory epithelium of the utricles. (**D1**) The high magnification of picture D shows the EdU+/Sox2+ cells in the utricular sensory epithelium of the Notch1-flox (f/f) Catnb-flox (exon3) (f/+) Sox2-CreER (+/−) group. (**E**) The histograms show the differences in the number of EdU+/Sox2+ cells between these groups. The cells were counted per 100 μm × 100 μm in the striolar or extrastriolar region of the utricles. In the Notch1-flox (f/f); Catnb-flox (exon3) (f/+); Sox2-CreER (+/−) mice, there were significantly more EdU+/Sox2+ SCs in the utricle sensory epithelium than in the other groups (*p < 0.05). (F) The histograms show the differences in the number of SCs between these groups. The cells were counted per 100 μm × 100 μm in the striolar or extrastriolar region of the utricles. The total number of SCs was also significantly increased compared to other groups (*p < 0.05, **p < 0.01, ***p < 0.001).

Similar to the above experiments, there were no obvious EdU+/Sox2+ SCs in the utricular sensory epithelium in the control group (Fig. 4A,E, Supplemental Table 4). In the Notch1-flox (f/f) Sox2-CreER (+/−) groups, there were some EdU+/Sox2+ SCs (Fig. 4B,E, Supplemental Table 4). In Catnb-flox (exon3) (f/+) Sox2-CreER (+/−) mouse utricles, there were only a few EdU+/Sox2+ SCs in the sensory epithelium (Fig. 4C,E, Supplemental Table 4). In the Notch1-flox (f/f); Catnb-flox (exon3) (f/+); Sox2-CreER (+/−) mice, there were significantly more EdU+/Sox2+ SCs in the utricular sensory epithelium than in the other groups (Fig. 4D,E, Supplemental Table 4, *p* < 0.05) and the total number of SCs was also significantly increased compared to the other groups (Fig. 4F, Supplemental Table 4, *p* < 0.05).

These results demonstrated the cumulative effects of Notch inhibition and Wnt activation in promoting the SC proliferation after gentamicin-induced HC damage. Simultaneous Notch inhibition and Wnt activation can significantly promote SC proliferation in neonatal mouse utricles after HC damage.

### After HC ablation, simultaneous Notch inhibition and Wnt activation increases the HC number and promotes the mitotic regeneration of HCs in neonatal mouse utricles *in vitro*

Similarly as above, we dissected out the P3 utricles, cultured them with 2 mM gentamicin for 48 hours, and then treated them with 5 μM DAPT and/or 10 μM QS11 for 7 days. All cultures were treated with 5 μM EdU together with the DAPT and QS11 for the first 24 hours.

**Figure 5:**
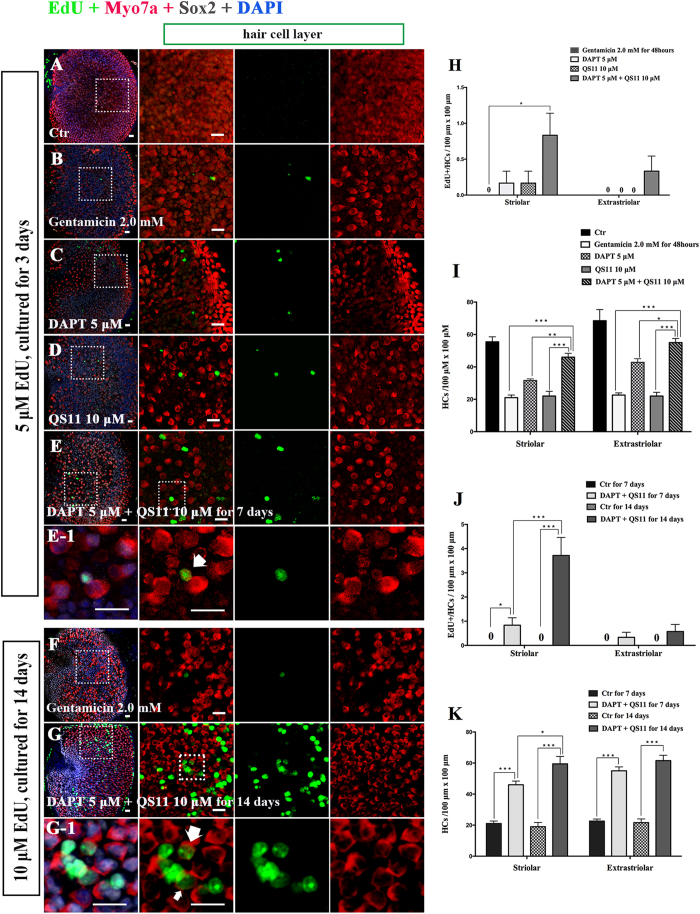
The P3 mouse utricles were treated with gentamicin for 48 hours then treated with DAPT and QS11 separately or in combination for 7 days or 14 days. (Scale bar = 10 μm) (**A**) The untreated P3 mouse utricles were cultured for 7 days. (**B**) The P3 mouse utricles were treated with gentamicin for 48 hours and cultured for 7 days. (**C**) When utricles were treated with DAPT for 7 days after HC loss, there were few EdU+/Myo7a+ cells in the utricles. (**D**) When utricles were treated with QS11 for 7 days after being treated with gentamicin, there were few EdU+/Myo7a+ cells in the utricles. (**E**) When utricles were treated with a combination of DAPT + QS11 for 7 days after being treated with gentamicin, there were some EdU+/Myo7a+ cells, and the number of HCs was greater than the other groups (*p* < 0.05). (**E1**) The high magnifications of picture E show the EdU+/Myo7a+ cell in the utricle. (**H**) The histograms show differences in the number of EdU+/HCs between these groups cultured for 7 days. (**I**) The histograms show differences in the number of HCs between these groups cultured for 7 days. **(F)** The P3 mouse utricles were treated with gentamicin for 48 hours and cultured for 14 days. **(G)** In the cultured utricles co-treated with DAPT and QS11 and supplied with 10 μM EdU for 14 days, there were some EdU+/Myo7a+ cells in the utricles. **(G-1)** The high magnifications of picture image G show the EdU+/Myo7a+ cells in the utricles. **(J)** The histograms show that the number of EdU+/Myo7a+ cells in the 14-day culture group was significantly greater than in the 7-day culture group. **(K)** The histograms show the differences in the number of HCs between the utricles co-treated with DAPT and QS11 for 7 days and those treated for 14 days. The cells were counted per 100 μm × 100 μm in the striolar or extrastriolar region of the utricles (*p < 0.05, **p < 0.01, ***p < 0.001).

We found that the number of HCs was greater compared to the control group after HC ablation in the 5μM DAPT-treated utricles, (*p* < 0.05) (Fig. 5C,I, Supplemental Table 3), and we found very few EdU+/Myo7a+ cells (Fig. 5C,H, Supplemental Table 3). In the QS11-treated utricles, we found that the number of HCs was similar to the control group (*p* > 0.05) (Fig. 5D,I, Supplemental Table 3), and we found very few EdU+/ Myo7a+ cells (Fig. 5D,H, Supplemental Table 3). In the DAPT and QS11 co-treated group, we found a significantly greater number of HCs than in the gentamicin and the single-treated groups (*p* < 0.05) (Fig. 5E,I, Supplemental Table 3), and the number of EdU+/Myo7a+ cells in the striolar region of DAPT and QS11 co-treated utricles was relatively greater than the gentamicin-treated group (*p* < 0.05) (Fig. 5E,H, Supplemental Table 3).

In order to trace the proliferated SCs, we cultured the utricles with 5 μM DAPT and 10 μM QS11 for 14 days after being damaged with gentamicin, and we supplied them with 10 μM EdU every day. We identified many more EdU+/Myo7a+ cells in the utricles after 14 days of treatment, and most of them were located in the striolar region (Fig. 5G,J, Supplemental Table 3). Compared with the results from 7 days of co-treatment with DAPT and QS11 after HC loss, the number of HCs increased significantly (Fig. 5G,K, Supplemental Table 3S, *p* < 0.05).

To further study the effects of co-regulation of Wnt and Notch signaling *ex vivo*, we also took advantage of Sox2-CreER, Notch1-flox (exon 1), and β-catenin-flox (exon 3) mice. The P3 utricles were isolated and cultured with 2 mM gentamicin for 48 h. Tamoxifen was then used to active the Cre recombinase and the utricles continued to be cultured for another 7 days.

**Figure 6:**
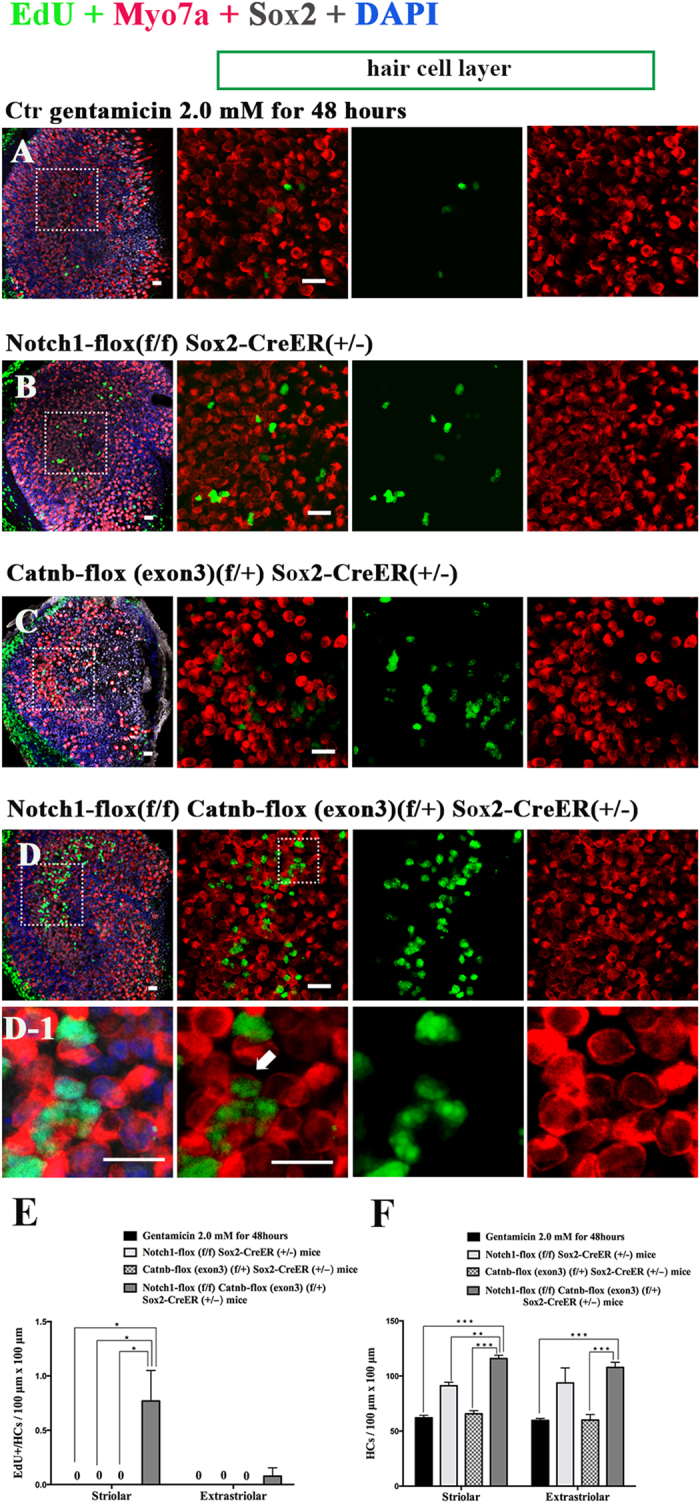
Sox2-CreER and Notch1-flox (exon 1) mice were mated with Catnb-flox (exon 3) mice to generate pups. The P3 mouse utricles were dissected and cultured with 2 mM gentamicin for 48 hours to damage the HCs of the utricles. The cultured utricles were treated with tamoxifen and cultured for an additional 7 days. (Scale bar = 10 μm) (**A**) In the control group, there were no obvious EdU+/ Myo7a+ cells in the sensory epithelium of the utricles. The number of HCs did not change significantly. (**B**) In the Notch1-flox (f/f) Sox2-CreER (+/−) mouse utricles, the number of HCs was increased compared to the control group (*p* < 0.05). (**C**) In the Catnb-flox (exon3) (f/+) Sox2-CreER (+/−) mouse utricles, the number of HCs did not change significantly. (**D**) In the Notch1-flox (f/f) Catnb-flox (exon3) (f/+) Sox2-CreER (+/−) group, there were some EdU+/Myo7a+ cells in the sensory epithelium of the utricles. The number of HCs also increased significantly. (**p < 0.01, ***p < 0.001). (**D1**) The high magnification of image D shows one of the EdU+/Myo7a+ cells in the utricular sensory epithelium of the Notch1-flox (f/f) Catnb-flox (exon3) (f/+) Sox2-CreER (+/−) mice. (**E**) The histograms show the differences in the number of EdU+/HCs between these groups. The cells were counted per 100 μm × 100 μm in the striolar or extrastriolar region of the utricles. No EdU+/Myo7a+ cells were observed in control and single-regulated groups. In the Notch1-flox (f/f) Catnb-flox (exon3) (f/+) Sox2-CreER (+/−) mice, we observed some EdU+/Myo7a+ cells in the utricular sensory epithelium. (*p < 0.05) (**F**) The histograms show the differences in the number of HCs between these groups. The cells were counted per 100 μm × 100 μm in the striolar or extrastriolar region of the utricles. The number of HCs in the Notch1-flox (f/f) Catnb-flox (exon3) (f/+) Sox2-CreER (+/−) mice utricles was greater than the other group (**p < 0.01, ***p < 0.001).

Similarly to the results from wild-type mice, the number of HCs in the Notch1-flox (f/f) Sox2-CreER (+/−) mice was greater than in the controls (*p* < 0.05) (Fig. 6B,F, Supplemental Table 4). In the Catnb-flox (exon 3) (f/+) Sox2-CreER (+/−) group, the number of HCs was not significantly different compared to controls (*p* > 0.05) (Fig. 6C,F, Table 4). In the Notch1-flox (f/f) Catnb-flox (exon3) (f/+) Sox2-CreER (+/−) mice, we found that there were significantly more HCs than all other groups (*p* > 0.05) (Fig. 6D,F, Supplemental Table 4). No EdU+/Myo7a+ cells were observed in the control and single-regulated groups. In the Notch1-flox (f/f) Catnb-flox (exon3) (f/+) Sox2-CreER (+/−) mice, we observed some EdU+/Myo7a+ cells in the utricular sensory epithelium. Most of these EdU+/Myo7a+ cells were found in the striolar region of the utricles, and very few of them were in the extrastriolar region (Fig. 6D,E, Table 4).

All of these results demonstrated that inhibiting Notch signaling can induce mitotic regeneration of HCs in damaged utricles and that Wnt activation can enhance the Notch-induced mitotic HC regeneration. Thus simultaneous Wnt activation and Notch inhibition can significantly promote the mitotic regeneration of HCs in damaged utricles.

### Simultaneous Notch inhibition and Wnt activation promotes the proliferation of SCs in adult mouse utricles *in vitro*

**Figure 7:**
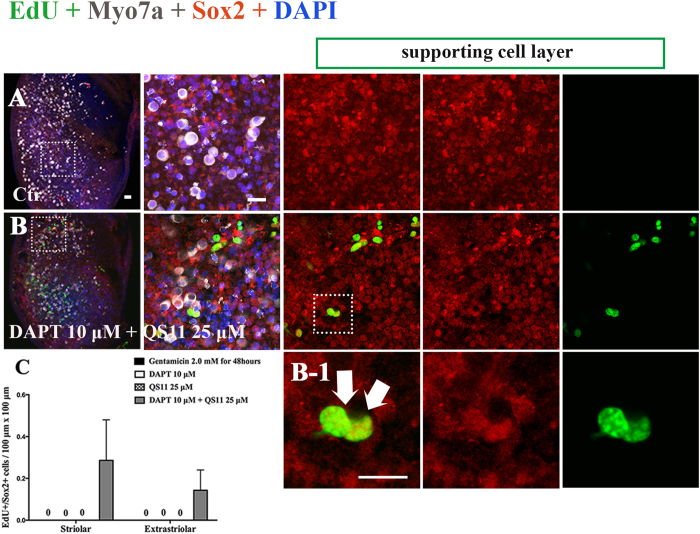
Cultured adult (2 months old) mouse cultured utricles were treated with 1 mM gentamicin for 24 hours then treated with 10 μM DAPT and 25 μM QS11 for 7 days. There were some EdU+/Sox2+ cells in the DAPT and QS11 co-treated adult mouse utricles. (Scale bar = 10 μm).

To further investigate whether Notch inhibition and Wnt activation can also induce SC proliferation in the utricles of adult mice after HC ablation, we isolated P60 (2-month old) mouse utricles and cultured them with 1 mM gentamicin for 24 hours and then treated them with 10 μM DAPT and/or 25 μM QS11 for 7 days. In the DAPT-only and QS11-only groups, we failed to find any EdU+/Sox2+ cells in the utricular sensory epithelium. We could occasionally find some EdU+/Sox2+ cells in the utricular sensory epithelium in the DAPT and QS11 co-treated group (Fig. 7, Supplemental Table 5), but no EdU+/Myo7a+ cells were observed in any of the four groups. These results demonstrated that after gentamicin-induced HC damage, simultaneous Notch inhibition and Wnt activation could promote SC proliferation even in the adult mouse utricle *in vitro.*

## Discussion

Non-mammalian vertebrates can spontaneously regenerate sensory HCs to recover hearing and balance function after HC damage by ototoxic drugs or acoustic trauma^3^,^36^. In the mammalian inner ear, however, sensory HCs have very limited regeneration ability after damage, which means that hearing and balance impairments are permanent^37^. Although the mammalian utricle has some ability to regenerate HCs spontaneously, which could lead to at least partial recovery of the peripheral vestibular function^38^,^39^, the quantity and quality of the spontaneously regenerated HCs are not sufficient to fully restore balance after damage to the inner ear.

The Notch signaling pathway plays important roles during the development of the inner ear^24^. Through lateral induction effects, mediated by Jagged1/Notch1, Notch signaling specifies sensory progenitors, and the overactivation of NICD (the Notch1 intracellular domain) in Pax2+ otic cells increases the size of the otic placode. On the other hand, through lateral inhibition effects, the HCs and SCs are precisely arranged in a mosaic distribution pattern within the organ of Corti^40^, and the loss of Notch signaling generates supernumerary HCs^26^,^41^,^42^ in the cochlea and in the utricle^8^. After HC ablation, Notch signaling is up regulated in the mouse cochlea^43^, and the inhibition of Notch signaling by a γ-secretase inhibitor can induce the regeneration of cochlear HCs and can lead to partial hearing recovery after acoustic trauma^26^. Many previous studies have shown that the HCs generated through Notch inhibition are mainly from direct trans-differentiation of SCs^8^, and it has been reported that the inhibition of Notch signaling triggers the proliferation of SCs in the cochlea during the embryonic stage. Our recent study demonstrated that Notch signaling serves as a negative regulator that inhibits the proliferation of Lgr5+ SCs as well as the subsequent mitotic generation of HCs in the mouse cochlea. In the current study, we did not observe as many proliferating SCs as we expected after Notch inhibition in neonatal utricles, and the hair cells mainly came from transdifferentiation at the expense of SCs.

Lgr5+ cells, which are responsive to Wnt signaling, have recently been identified as the progenitors in the mouse cochlea^29^,^44^,^45^. By overexpressing β-catenin in SCs, Lgr5+ SCs can proliferate and form cell foci, and a small portion of these proliferated cells can generate HCs in the neonatal mouse cochlea^29^,^46^. Lgr5 is no longer expressed in the vestibular epithelia after birth, but Lgr5+ supporting cells can still be recruited to the striolar region of the neonatal mouse utricle after HC loss^47^. By overexpressing β-catenin in Sox2+ cells with or without HC loss, we observed the proliferation of SCs, which were mainly located in striolar region, but very few of them could transdifferentiated into HCs even after long-term culture.

In the current study, we achieved the proliferation of SCs as well as the mitotic generation of HCs by inhibiting Notch and activating Wnt signaling in the neonatal mouse utricle with or without HC loss, and this further suggested that the crosstalk between Wnt and Notch signaling plays an important role during HC generation in the mouse utricle.

The Notch and Wnt signaling pathways engage in crosstalk when regulating cell proliferation and differentiation in a variety of tissues^43^,^48^,^49^,^50^,^51^. In the intestinal epithelium, crosstalk between the Wnt and Notch signaling plays an important role in regulating and maintaining the balance between proliferation and differentiation of epithelial stem cells and immature progenitor cells^49^,^50^. It has also been shown that Notch4 promotes gastric cancer growth through activation of Wnt1/β-catenin signaling^51^. Another recent study showed that the Notch inhibitor DAPT regulates human hair follicle stem cell proliferation and differentiation through regulation of p21 and Wnt-10b^52^. The crosstalk between the Notch and Wnt signaling pathways is also involved in the pathogenesis of hepatocellular carcinoma. Wnt signaling occurs downstream of the Notch pathway in regulating the proliferation of L02/HBx cells, and Notch1 promotes hepatitis B virus X protein-induced hepatocarcinogenesis via the Wnt/β-catenin pathway^53^. Despite all of this previous research, however, the role of Notch and Wnt crosstalk in inner ear sensory cell proliferation and regeneration has remained unclear. In the inner ear, Notch inhibition can promote HC differentiation from inner ear progenitors/stem cells^54^, and Wnt activation can promote the proliferation of inner ear progenitors/stem cells in the neonatal mouse cochlea^29^. A recent study showed that HC differentiation induced by Notch inhibition was due to overcoming the lateral inhibition of converting SCs into HCs and was also dependent on the Wnt signaling pathway^55^. In addition, our previous study implied that Notch inhibition initiated the proliferation of SCs and mitotic regeneration of HCs by activating the canonical Wnt signaling pathway, while the inhibition of Wnt signaling decreased the SC proliferation and mitotic regeneration of HCs initiated by Notch inhibition^56^. In this study, similar to previous reports, we also found that Notch inhibition induced SC proliferation and HC regeneration. When we simultaneously inhibited Notch signaling together with activation of Wnt signaling, we found significantly greater SC proliferation and mitotic HC regeneration than only inhibiting Notch or only activating Wnt *in vitro*, and the simultaneous inhibition of Notch and activation of Wnt also significantly increased the total number of HCs and the number of mitotically regenerated HCs. These results suggest that the proliferation of SCs and mitotic regeneration of HCs triggered by Notch inhibition were not only dependent on the Wnt signaling pathway, but were promoted by the up-regulation of the Wnt signaling pathway.

In this study, we found that the number of EdU+/Sox2+ SCs as well as the number of EdU+/Myo7a+ HCs increased during the extended culture period, which suggested that there is a cumulative effect or dose-dependent effect of chemical reagent treatment during the generation of HCs in mouse utricles and that the conversion from SCs to HCs and the maturation of HCs are time-consuming processes. We found that only 9.17% (striolar region) and 1.43% (extrastriolar region) of the HCs are EdU+ /Myo7a+ cells in co-treated utricles after 14 days culture and that only 18.73% (striolar region) and 19.86% (extrastriolar region) of the SCs could transdifferentatiate into HCs, which suggests that direct transdifferentiation is the main source of HC generation and that the strategies for promoting the conversion from proliferated SCs into HCs need to be further develpoed.

It has been reported that the spontaneous proliferation of SCs mainly occurs in the damaged region of the utricle after HC loss, and the integrity of the sensory epithelium might suppress the proliferation of SCs and the extrusion of damaged HCs. In current study, we observed mitotic HC generation by inhibiting Notch and activating Wnt signaling in cultured mouse utricles without HC loss, which suggested that the joint effect of Notch inhibition and Wnt activation could overcome the brakes on sensory cell proliferation and regeneration that maintain the integrity of the vestibular sensory epithelium. Compared with the undamaged utricles co-treated with DAPT and QS11, we did not observe significantly more EdU+/Sox2+ or Myo7a+/EdU+ cells in the damaged sensory epithelium co-treated with DAPT and QS11, which is different from our expectation that HC loss might serve as another promoting factor for HC regeneration. The limited proliferation and HC regeneration might be due to the possible injury to supporting cells induced by gentamicin^57^,^58^. Another mouse model in which HCs can be killed more specifically without damage to the SCs should be developed for further investigations.

In the mouse utricle, the difference between striolar SCs and extrastriolar SCs remains largely uninvestigated. A recent study reported that after HC ablation, the expression of Lgr5, a Wnt downstream target gene, is re-activated specifically in a subset of SCs in the striolar region, but not in the extrastriolar region, and these damage-activated Lgr5+ SCs act as the HC progenitors in the mouse utricle^47^. In our present study, we found that the striolar region of the utricle has greater SC proliferation and HC mitotic regeneration ability than the extrastriolar region after simultaneous Notch inhibition and Wnt activation.

Previous studies reported that the utricle’s capacity for SC proliferation and HC regeneration decreases with age^59^,^60^. In this study, we also found that with increased age the SC proliferation and HC mitotic regeneration induced by Notch inhibition and β-catenin up-regulation were significantly decreased. In adult utricles (P60), no mitotically regenerated HCs were observed in any group, and no SC proliferation was detected in control or single-regulated groups. However, in adult utricles, we still could find some proliferated SCs after simultaneous Notch inhibition and Wnt up-regulation after HC ablation, and this suggests that co-regulation of Notch and Wnt signaling promotes significant SC proliferation even in the adult utricle.

In summary, we report here that simultaneous Notch inhibition and Wnt activation promotes significant SC proliferation and mitotic HC regeneration with or without HC damage. Thus, the appropriate co-regulation of the Notch and Wnt signaling pathways might provide a new route to mitotically regenerate more HCs while preserving the SCs after damage in the mammalian inner ear and thereby recover hearing and balance function.

## Methods

### Animals

Wild-type neonatal (postnatal day (P) 3 and adult (6 weeks to 2 months old) C57BL/6j mice were from Fudan Medical School (Shanghai, China), Notch1-flox (exon 1) and Catnb-flox (exon 3) mice were purchased from the Jackson Laboratories, and Sox2-Cre-ER mice were a gift from Dr. Konrad Hochedlinger of Harvard University. The care and use of animals were approved by the Institutional Animal Care and Use Committee of Fudan University in compliance with the NIH guidelines for the care and use of laboratory animals.

### Organotypic culture of wild-type mouse utricles

The mice were euthanized by carbon dioxide asphyxiation and decapitated, and their heads were placed in 75% ethanol and quickly transferred to chilled Hanks’ balanced salt solution (HBSS). The temporal bones were dissected out, and the utricles were isolated from the temporal bone using sterile procedures in ice-cold HBSS. The otoconia on the utricles were gently removed with fine forceps.

Explants of utricles were placed intact on polylysine-coated cover glasses (Sigma, St. Louis, MO, USA) and maintained in four-well culture dishes (Greiner Bio-One, Frickenhausen, Germany) in Dulbecco’s modified Eagle’s medium (DMEM) and F12 medium supplemented with N2 and B27 (Invitrogen/GIBCO/BRL, Carlsbad, CA) and 50 I U/mL penicillin (Sigma).

### Treatment of utricle cultures

Utricle cultures were incubated with culture medium supplemented with 10% FBS for about 2 hours for adhering. The cultures were then treated with DAPT (γ-secretase inhibitor IX, N- [N- (3,5- difluorophenacetyl) -l-alanyl] -S-phenylglycine t-butyl ester, EMD, Gibbstown, NJ) and QS11 ((2S)-2-[2-(Indan-5-yloxy)-9-(1,1′-biphenyl-4-yl) methyl]-9H-purin-6- ylamino)-3-phenyl-propan-1-ol, Tocris Biosciences, USA), which modulates ARF-GTP levels and synergizes with the Wnt/β-catenin signaling pathway to upregulate β-catenin nuclear translocation. Both compounds were initially dissolved in sterile dimethyl sulfoxide (DMSO, Sigma) to a concentration of 20 mM and stored in aliquots at −20 °C before dilution in culture medium to their final concentration immediately before use. The cultured utricles were treated with 5 μM DAPT and/or 10 μM QS11 for 3 days, and 5 μM EdU was added for the first 24 hours of the culture period to label the proliferating cells. One group of utricle cultures was treated with 5 μM DAPT and 10 μM QS11 supplemented with 10 μM EdU for 14 days, and fresh EdU was added once a day during the culture period to label the proliferating cells. The tissues were incubated at 37 °C in a humidified atmosphere of 95% air and 5% CO_2_.

Another group of utricle cultures was first treated with gentamicin (Invitrogen/GIBCO) to damage the HCs. After washing with fresh culture medium, the utricles were treated with 5 μM DAPT and/or 10 μM QS11 for 7 more days, and 5 μM EdU was added for the first 24 hours. One group of utricles was cultured with 5 μM DAPT and 10 μM QS11 together with 10 μM EdU for 14 days, and fresh EdU was delivered once a day. Control cultures were cultured in 0.1% DMSO or PBS.

### Treatment of Notch1 knockout and β-catenin over-expressing mouse utricles

Sox2-CreER and Notch1-flox (exon 1) mice were mated with Catnb-flox (exon3) mice to generate Sox2-CreER; Notch1-flox (exon 1); Catnb-flox (exon3) mice. Littermates without Notch1-flox (exon 1) or without Catnb-flox (exon3) were used as controls. Tamoxifen (2 mg/25 g, Sigma) was used to activate the Cre recombinase, which was given to the female mice by intraperitoneal injection. The mother transferred the tamoxifen to the pups via her milk. EdU (50 mg/kg, intraperitoneal injection, Invitrogen) was given to pups twice a day for 7 days. The neonatal pups were sacrificed at 7 days, and their utricles were dissected out for immunohistochemistry processing. The mice were genotyped after sacrifice, and the genotyping primers are described in Supplemental Table 1.

We also dissected the utricles from neonatal (P3) mice to explant them onto polylysine-coated cover glasses (Sigma), and they were maintained in four-well culture dishes (Greiner Bio-One) with culture medium. The tissues were incubated at 37 °C in a humidified atmosphere of 95% air and 5% CO_2_. All the pups were genotyped after they were sacrificed.

After 2 hours of adhering to the cover glass, the utricles were given 2 mM gentamicin (Invitrogen/ GIBCO) for 48 hours. The cultured tissues were washed thoroughly with fresh medium, and they were treated with 1 μM 4OH-tamoxifen (Sigma) for 2 days and then cultured in medium supplemented with 10 μM EdU for 7 more days.

### Immunohistochemistry

The cultures were harvested and fixed for 30 minutes at room temperature with 4% paraformaldehyde in 0.1 M phosphate buffer and then thoroughly rinsed with 0.01 M PBS. After being permeabilized in 0.5% Triton X-100 in PBS (PBST) for 30 minutes at room temperature, the proliferating cells were labeled with EdU (Click-iT EdU Alexa Fluor 488 imaging kit, Invitrogen) for 30 minutes according to the manufacturer’s protocol. The specimens were blocked with 10% horse serum in PBST for 30 minutes. The HCs were labeled with the rabbit antibody against Myo7a (1:500 dilution; Proteus Biosciences) and the goat polyclonal antibody against Sox2 (1:200 dilution; Santa Cruz Biotechnology) at 4 °C overnight.

After being washed with PBST to remove the unbound antibodies, the specimens were incubated with Alexa Fluor 594-conjugated donkey anti-rabbit (1:500 dilution; Invitrogen) and Alexa Fluor 647-conjugated donkey anti-goat (1:200 dilution; Invitrogen) secondary antibodies diluted in PBST for 1 hour at room temperature to visualize Myo7a and Sox2, respectively. The specimens were stained with 4′, 6-diamidino-2-phenylindole dihydrochloride (DAPI) for 5 minutes at room temperature to visualize the cell nuclei.

### Image acquisition and quantification

The fluorescence signals from the cultured utricles were visualized using a Nikon (Japan) Eclipse 80i microscope. The high-magnification fluorescent images were obtained with a Leica TCS SP5 scanning microscope (Wetzlar, Germany). Cells were counted manually on the stored images with the Image J software (Wayne Rosband, NIH, USA). The cells were counted from four randomly selected extrastriolar and striolar 100 μm × 100 μm regions per specimen. At least five samples in each group from three independent experiments were collected for statistical analysis. The cell counts for the control and treated groups were compared using Student’s *t*-test.

### Statistics

Statistical analyses were conducted using Microsoft Excel and GraphPad Prism software. All data were analyzed using a two-tailed, unpaired Student’s *t*-test when comparing two groups or with a one-way ANOVA followed by a Dunnett’s multiple comparisons test when comparing more than two groups. All data are expressed as either a percentage or as the mean ± SEM. *p*-values < 0.05 were considered statistically significant.

## Additional Information

**How to cite this article**: Wu, J. *et al*. Co-regulation of the Notch and Wnt signaling pathways promotes supporting cell proliferation and hair cell regeneration in mouse utricles. *Sci. Rep.* **6**, 29418; doi: 10.1038/srep29418 (2016).

## Supplementary Material

Supplementary Information
