# Supplementary material for: Co-regulation of the Notch and Wnt signaling pathways promotes supporting cell proliferation and hair cell regeneration in mouse utricles
Source: Sci Rep. 2016 Jul 20;6:29418. doi: 10.1038/srep29418 (PMC4951696; doi:10.1038/srep29418)
Supplement: Supplementary Information [file srep29418-s1.pdf]

Sup-Table 1. Primers used in the study.

Gene Name	Forward Primer	Revers Primer
Sox2creER	5'-gcggtctggcagtaaaaactatc-3'	5'-gtgaaacagcattgctgtcactt-3'
	5'-ctaggccacagaattgaaagatct-3'	5'-gtaggtggaaattctagcatcatcc-3'
Notch1	5'-tgccctttccttaaaagtgg-3'	5'-gcctactccgacacccaata-3'
Beta-catenin OE	5'-aactggcttttggtgtcggg-3'	5'-tcggtggcttgctgattatttc-3'

Sup-Table 2. The number of EdU+/Sox2+ cells, SCs, EdU+ HCs, and HCs (mean ± SEM) in the treated utricles (P3 mouse) cultured for 3 days or 14 days.

	Striolar region (per 100x100 μm^2 region) (mean ± SEM)				Extrastriolar region (per 100x100 μm^2 region) (mean ± SEM)			
**Treatment**	EdU+/Sox2+ cells	SCs	EdU+/HCs	HCs	EdU+/Sox2+ cells	SCs	EdU+/HCs	HCs
Control cultured for 3 days	0.25 ± 0.25	191.52 ± 7.57	0	55.00 ± 3.56	0	226.83 ± 7.10	0	67.75 ± 4.15
DAPT treated for 3 days	2.00 ± 0.33	209.00 ± 3.61	0.25 ± 0.16	93.43 ± 3.52	1.13 ± 0.30	223.50 ± 7.73	0	108.43 ± 8.36
QS11 treated for 3 days	2.33 ± 0.50	192.44 ± 10.46	0.17 ± 0.11	67.60 ± 3.52	1.17 ± 0.31	224.80 ± 11.53	0	76.40 ± 5.71
DAPT and QS11 co-treated for 3 days	4.00 ± 0.54	213.90 ± 6.59	0.50 ± 0.27	127.50 ± 9.70	2.43 ± 0.53	223.89 ± 6.30	0.25 ± 0.16	143.67 ± 6.65
Control cultured for 14 days	0.75 ± 0.48	188.25 ± 11.95	0	53.33 ± 3.74	0.25 ± 0.25	218.50 ± 11.46	0	63.00 ± 3.87
DAPT and QS11 co-treated for 14 days	20.63 ± 3.41	233.30 ± 10.37	3.57 ± 0.65	158.71 ± 9.59	2.75 ± 0.71	236.11 ± 13.60	0.43 ± 0.20	158.60 ± 3.71

Sup-Table 3. After HC ablation, the number of EdU+/Sox2+ cells, SCs, EdU+ HCs, and HCs (mean ± SEM) in the treated utricles (P3 mouse) cultured for 3 days or 14 days.

	Striolar region (per 100x100 μm² region) (mean ± SEM)				Extrastriolar region (per 100x100 μm² region) (mean ± SEM)			
**Treatment**	EdU+/Sox2+ cells	SCs	EdU+/HCs	HCs	EdU+/Sox2+ cells	SCs	EdU+/HCs	HCs
Gentamicin treated and cultured for 7 days	0.75 ± 0.48	206.17 ± 10.97	0	21.00 ± 1.64	0.25 ± 0.25	234.33 ± 8.43	0	22.60 ± 1.36
DAPT treated for 7 days	1.67 ± 0.67	217.40 ± 9.70	0.17 ± 0.17	31.60 ± 1.03	1.67 ± 0.80	240.60 ± 16.04	0	42.80 ± 2.27
QS11 treated for 7 days	1.17 ± 0.48	222.67 ± 9.31	0.17 ± 0.17	22.00 ± 2.93	1.17 ± 0.40	239.00 ± 8.34	0	22.00 ± 2.31
DAPT and QS11 co-treated for 7 days	3.33 ± 0.67	247.50 ± 13.82	0.83 ± 0.31	46.00 ± 2.41	2.33 ± 0.56	256.80 ± 14.04	0.33 ± 0.21	55.00 ± 2.51
Gentamicin treated and cultured for 14 days	1.00 ± 0.41	195.83 ± 10.92	0	19.00 ± 2.70	0.50 ± 0.29	221.50 ± 14.27	0	21.60 ± 2.38
DAPT and QS11 co-treated for 14 days	16.10 ± 4.30	277.50 ± 8.59	3.71 ± 0.75	59.46 ± 4.81	2.30 ± 0.63	280.25 ± 8.48	0.57 ± 0.30	61.55± 3.47

Sup-Table 4. The number of EdU+/Sox2+ cells, SCs, EdU+/HCs, and HCs (mean ± SEM) in the transgenic mouse utricles (P3 mouse).

	**Striolar region (per 100x100 µm^2 region) (mean ± SEM)**				**Extrastriolar region (per 100x100 µm^2 region) (mean ± SEM)**			
**Treatment**	EdU+/Sox2+ cells	SCs	EdU+/HCs	HCs	EdU+/Sox2+ cells	SCs	EdU+/HCs	HCs
Gentamicin treated utricles	0.25 ± 0.25	166.60 ± 15.99	0	62.00 ± 2.31	1.00 ± 0.58	164.17 ± 10.57	0	59.63 ± 1.77
Notch1-flox (f/f) Sox2-CreER (+/-) mice	5.17 ± 1.54	151.83 ± 4.24	0	91.17 ± 3.10	1.50 ± 0.96	158.80 ± 8.52	0	93.67 ± 13.68
Catnb-flox (exon3) (f/+) Sox2-CreER (+/−) mice	4.33 ± 1.45	158.00 ± 4.73	0	65.67 ± 2.91	1.33 ± 0.88	158.67 ± 3.18	0	60.00 ± 5.00
Notch1-flox (f/f) Catnb-flox (exon3) (f/+) Sox2-CreER (+/−) mice	22.86 ± 7.20	204.17 ± 3.53	0.77 ± 0.28	115.78 ± 3.14	10.33 ± 2.71	212.50 ± 9.10	0.08 ± 0.08	107.78 ± 4.67

Sup-Table 5. The number of EdU+/Sox2+ cells (mean ± SEM) in the treated utricles (P60 mouse).

	**Striolar region (per 100x100 µm^2 region) (mean ± SEM)**	**Extrastriolar region (per 100x100 µm^2 region) (mean ± SEM)**
**Treatment**	EdU+/Sox2+ cells	EdU+/Sox2+ cells
Gentamicin treated utricles	0	0
DAPT treated utricles	0	0
QS11 treated utricles	0	0
DAPT and QS11 co-treated utricles	0.29 ± 0.19	0.14 ± 0.10
